# Relugolix vs. Leuprolide Effects on Castration Resistance-Free Survival from the Phase 3 HERO Study in Men with Advanced Prostate Cancer

**DOI:** 10.3390/cancers15194854

**Published:** 2023-10-05

**Authors:** Fred Saad, Daniel J. George, Michael S. Cookson, Daniel R. Saltzstein, Ronald Tutrone, Alberto Bossi, Bruce Brown, Bryan Selby, Sophia Lu, Bertrand Tombal, Neal D. Shore

**Affiliations:** 1 University of Montreal Hospital Centre, Montreal, QC H2X 3E4, Canada; 2 Duke Cancer Institute Center for Prostate and Urologic Cancers, Duke University, Durham, NC 27710, USA; daniel.george@duke.edu; 3 Department of Urology, The University of Oklahoma Health Sciences Center, Oklahoma City, OK 73104, USA; michael-cookson@ouhsc.edu; 4 Urology San Antonio, San Antonio, TX 78229, USA; daniel.saltzstein@urologysa.com; 5 Chesapeake Urology, Towson, MD 21204, USA; rtutrone@uniteduro.com; 6 Department of Radiation Oncology, Gustave Roussy Cancer Institute, 94805 Villejuif, France; alberto.bossi@gustaveroussy.fr; 7 Myovant Sciences, Inc., Brisbane, CA 94005, USA; brucebrown91@gmail.com (B.B.); bds_mv@yahoo.com (B.S.);; 8 Institut de Recherche Clinique, Université Catholique de Louvain, B-1348 Brussels, Belgium; bertrand.tombal@saintluc.uclouvain.be; 9 Carolina Urologic Research Center, Myrtle Beach, SC 29572, USA; nshore@auclinics.com

**Keywords:** relugolix, leuprolide, castration resistance-free survival, advanced prostate cancer

## Abstract

**Simple Summary:**

An advanced prostate cancer research study known as HERO compared the ability of the medications relugolix and leuprolide to lower testosterone. The goal was to lower the testosterone to sustained castration levels, which is defined as below 50 ng/dL. This analysis evaluated how long an individual’s disease progressed while their testosterone remained at castration levels during the study. This analysis is called castration resistance-free survival (CRFS) and compared men receiving relugolix or leuprolide in two populations: the group of individuals with metastatic disease (or disease that has progressed beyond the prostate) and the overall group of individuals enrolled in the study (that is those with and those without metastatic disease). This analysis showed that CRFS for relugolix and the standard-of-care leuprolide were the same in the population of men with metastatic disease as well as in the overall population of the HERO study.

**Abstract:**

**Background:** Relugolix is an oral GnRH receptor antagonist approved for men with advanced prostate cancer. Relugolix treatment has demonstrated an ability to lower testosterone to sustained castration levels in the phase 4 HERO study. Herein, we describe the results of a secondary endpoint of castration resistance-free survival (CRFS) during 48 weeks of treatment and profile patients with castration-resistant prostate cancer (CRPC). **Methods:** Subjects were 2:1 randomized to either relugolix 120 mg orally once daily (after a single 360 mg loading dose) or 3-monthly injections of leuprolide for 48 weeks. CRFS, defined as the time from the date of first dose to the date of confirmed prostate-specific antigen progression while castrated or death due to any reason was conducted in the metastatic disease population and the overall modified intention-to-treat (mITT) populations. **Results:** The CRFS analysis (mITT population) included 1074 men (relugolix: n = 717; leuprolide: n = 357) with advanced prostate cancer as well as 434 men (relugolix: n = 290; leuprolide: n = 144) with metastatic prostate cancer. In the metastatic disease populations, CRFS rates were 74.3% (95% CI: 68.6%, 79.2%) and 75.3% (95% CI: 66.7%, 81.9%) in the relugolix and leuprolide groups, respectively (hazard ratio: 1.03 [0.68, 1.57]; *p* = 0.84) at week 48. Results in the overall mITT population were similar to the metastatic population. No new safety findings were identified. **Conclusions:** In men with metastatic disease or in the overall population of the HERO study, CRFS assessed during the 48-week treatment with relugolix was not significantly different than standard-of-care leuprolide. Relugolix had similar efficacy for men with/without CRFS progression events.

## 1. Introduction

Gonadotropin-releasing hormone (GnRH) receptor agonists or antagonists given as androgen deprivation therapy (ADT) are a standard of care in advanced prostate cancer treatment [1,2,3,4,5,6,7]. Relugolix is a first-in-class, once-daily oral, and highly selective GnRH receptor antagonist, with an effective half-life of 25 h [8,9,10,11,12]. Relugolix was evaluated clinically in the pivotal phase 3 HERO study, where it showed sustained suppression of testosterone to castrate levels in 96.7% of patients. These results were superior to leuprolide (88.8%). The risk of major adverse cardiovascular events was lower with relugolix relative to leuprolide and was, overall, well tolerated [13].

Despite most patients responding initially to ADT, a significant proportion will progress to castration resistance despite effective castration [14]. Data from recent clinical studies indicate that patients with metastases usually respond between 7.4 to 18 months before castration resistance develops [15,16,17]. In contrast, patients with only biochemical recurrence may respond to ADT for 5 to 10 years, and only one-third will develop castration resistance [18]. 

Herein, we describe the results of the HERO study assessment of castration resistance-free survival (CRFS), a clinically relevant indicator of disease progression, in the overall modified intention-to-treat (mITT) population as well as the metastatic disease population. In addition, the profile of patients who experienced a CRFS progression event (i.e., those with castration-resistant prostate cancer [CRPC]) was evaluated.

## 2. Materials and Methods

### 2.1. Study Design

The HERO study was designed to evaluate the efficacy and safety of relugolix in men with advanced prostate cancer; details of the study design have been previously published (Clinical Trial ID: NCT03085095) [13]. Briefly, patients were randomized 2:1 to receive relugolix 120 mg orally once daily after a single loading dose of 360 mg or leuprolide injections every 12 weeks for 48 weeks. Randomization was stratified according to geographic region (North and South America, Europe, and Asia–Pacific region), the presence or absence of metastatic disease, and age (≤75 and >75 years).

The trial was approved by a central institutional review board, the institutional review board or independent ethics committee for each center and was conducted in accordance with the requirements of the regulatory authorities of each country and with the provisions of the Declaration of Helsinki and the Good Clinical Practice guidelines of the International Council for Harmonization. All the patients provided written informed consent.

### 2.2. Patients

Eligible patients were 18 years of age or older, were candidates for at least 1 year of continuous androgen deprivation therapy, and had histologically or cytologically confirmed adenocarcinoma of the prostate. To be eligible, patients had one of three clinical disease presentations: evidence of biochemical (PSA) or clinical relapse after local primary intervention with curative intent, newly diagnosed hormone-sensitive metastatic disease, or advanced localized disease unlikely to be cured by local primary intervention with curative intent. Patients with major adverse cardiovascular events (MACE) within 6 months before study initiation were excluded. MACE were defined as non-fatal myocardial infarction, non-fatal stroke, and death from any cause. Additional information on inclusion and exclusion criteria has been previously published [13]. 

### 2.3. Assessments and Endpoints

CRFS was a key secondary endpoint of the HERO study (not analyzed during primary analysis). CRFS was defined as the time from the date of the first dose to the date of confirmed prostate-specific antigen progression (defined by Prostate Cancer Clinical Trials Working Group 3; PGCW3) [19] while castrated or dead due to any reason, whichever occurs earlier. To assess CRFS, approximately 140 additional patients with metastatic disease population were planned to be enrolled, with the goal of including at least 390 patients with metastatic disease already enrolled in the original 915 patients (actual numbers: 434 metastatic patients and 1074 mITT patients treated for 48 weeks). PSA progression was confirmed as per PCWG3 criteria, which defines PSA progression as the date that an increase of 25% or more and an absolute increase of 2 ng/mL or more from the nadir are documented [19]. For patients who had an initial PSA increase during treatment, this must be confirmed by a second PSA increase 3 or more weeks later. A post-hoc multivariate Cox regression analysis was performed to assess which baseline characteristics were risk factors for CRFS events.

### 2.4. Statistical Analysis

CRFS in the 434 metastatic patients and the modified ITT population (1074 patients) were to be analyzed only at the time of the final analysis. Approximately 107 confirmed CRFS events (PSA progressions while castrated or death due to any cause) were needed (or approximately 390 metastatic patients would need to be enrolled) to detect a target hazard ratio of 0.55 (relugolix versus leuprolide acetate) with 85% power with a two-sided type I error of 5%, assuming a CRFS rate of 60% at 48 weeks for the control arm, an 18-month enrollment period, 12 months of additional follow-up, and a 15% dropout rate. 

For the analysis of the overall mITT population, it is anticipated that to observe approximately 149 confirmed CRFS events (PSA progression or death due to any cause) a total of approximately 1200 patients (metastatic or non-metastatic) randomized into the study would be needed. This is assuming an 18-month enrollment period, 12 months of additional follow-up, and a 10% dropout rate, the study will provide approximately an 85% power to detect a hazard ratio of 0.6 (relugolix versus leuprolide acetate) with a two-sided type I error of 5%.

A multivariate Cox regression model using baseline characteristics was used to predict the risk factors that impacted CRFS. The following baseline characteristics were evaluated as risk factors for CRFS in the model: Age (>65 y vs. <=65 y/also as a continuous variable); Metastatic disease; Disease stage at study entry; Clinical disease state presentation; Testosterone at baseline (>=250 ng/dL vs. <250 ng/dL/also as a continuous variable); PSA at baseline (>20 vs. <=20 ng/mL/also as a continuous variable); Gleason Score (<8 vs. >=8); Geographic region (NA vs. ROW); Race (white vs. others); Ethnicity (Hispanic vs. non-Hispanic); FSH level at baseline (>=11.71 IU/L vs. <11.71 IU/L [median from all patients]/also consider as continuous variable); Prior ADT use (Y vs. N); Life-style related risk (former/current smoker/heavy alcohol use/BMI > 30, combined or as separate risk factor); Cerebrovascular or cardiovascular risk in medical history; MACE history; and concomitant medications used at baseline (statin, anti-hypertension, and anti-thrombotic use, combined or as separate risk factor).

## 3. Results

### 3.1. Patients

Baseline demographics and clinical characteristics of the overall population and the metastatic disease population are summarized in Table 1. Overall, the CRFS analysis using the mITT population randomized 717 men to relugolix treatment and 357 men to leuprolide treatment. The metastatic disease analysis included 290 men who received relugolix and 144 men who received leuprolide. Baseline characteristics were generally similar between treatment groups and similar between the overall population and the metastatic disease population. Exceptions included a smaller percentage of people from North and South America and a larger percentage from Asia/Rest of the World from the metastatic disease population versus the overall population as well as a higher rate of bone metastases in the relugolix group.

### 3.2. Efficacy

**Figure 1:**
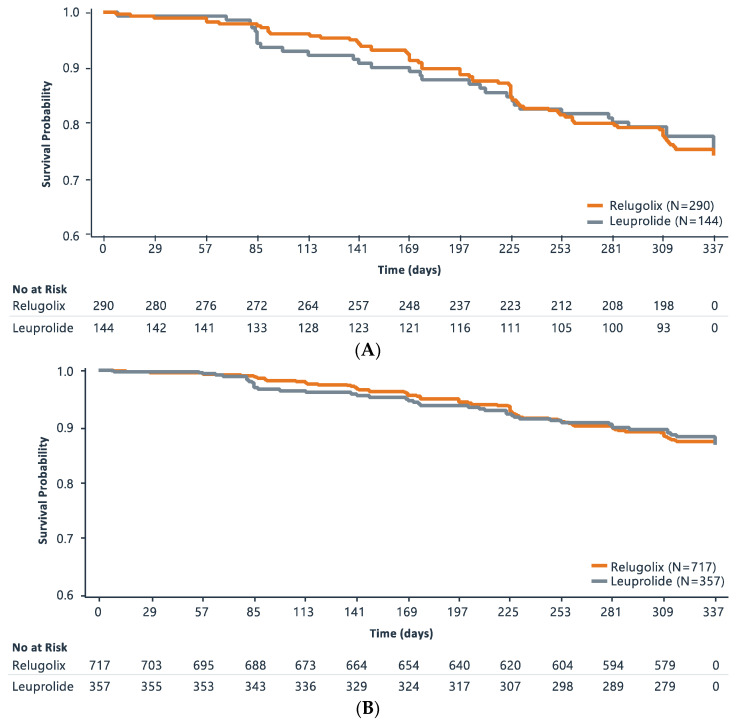
Kaplan–Meier Survival Curve of Castration-Free Survival Analysis in (**A**). Metastatic Patient Population and (**B**). Overall Population.

Kaplan–Meier curves of CRFS analysis for the metastatic population and the overall population are shown in Figure 1A and Figure 1B, respectively. In the metastatic disease population, events occurred in 68 men (23.4%) in the relugolix group and 32 men (22.2%) in the leuprolide group. The CRFS rates at 48 weeks were 74.3% (95% CI: 68.6%, 79.2%) and 75.3% (95% CI: 66.7%, 81.9%) in the relugolix and leuprolide groups, respectively. The difference between treatment groups of −0.96 (95% CI: −10.20, 8.28) in the metastatic disease population was not statistically different between the two treatment groups (hazard ratio: 1.03 (0.68, 1.57); *p* = 0.84. 

Results were similar between the two groups in the overall mITT population. Events occurred in 88 men (12.3%) in the relugolix group and 42 men (11.8%) in the leuprolide group. At 48 weeks in the overall population, the CRFS rates were 86.8% (95% CI: 84.0%, 89.2%) and 87.3% (95% CI: 83.2%, 90.5%) in the relugolix and leuprolide groups, respectively. In the overall population, the comparison was not statistically different between the two treatment groups (hazard ratio: 1.03 (0.72, 1.49); *p* = 0.89. 

Testosterone levels for the overall population at the time of CRFS progression event are shown in Table 2. During the study, 2 men in the leuprolide group were above the castration threshold (<50 ng/dL); both of these subjects died without attaining castration. All other men enrolled in the study were under 50 ng/dL at the time of their CRFS progression event. The final testosterone values on or before week 48 post-CRFS development show testosterone suppression was maintained. The sustained castration rate point estimates for 48 weeks with the relugolix and leuprolide groups were: 91.7% vs. 97.0% (difference: 5.3% (95% CI: −13.7%, 3.0%)) in men with a CRFS event and 97.0% vs. 88.4% (difference: 8.6% (95% CI: 4.7%, 12.4%)) in men who did not develop a CRFS event. 

A summary of baseline characteristics for the 88 men with a CRFS event during the study (CRPC population) as well as the overall population is provided in Table S1. Relative to the overall population, men who experienced a CRFS event were characterized at the study baseline by older age (median age: CRPC population: 72.0 years vs. overall population: 71.0 years; patients >75 years: 33.8% vs. 29.0%); and higher ECOG status (ECOG status ≥1: 25.4% vs. 13.8%); and higher PSA ≥20 (80.5% vs. 40.3%). Patients who experienced a CRFS event were also more likely to have metastatic disease at study entry (76.9% vs. 40.4%) and have multiple sites of metastasis at study entry (37.7% vs. 11.7%). A stepwise multivariate Cox regression analysis of potential baseline risk factors in all men was performed. Baseline characteristics that were associated with a CRFS event (*p* < 0.05) after stepwise selection were PSA ≥ 20 (*p* < 0.0001), and metastatic disease at baseline (*p* < 0.0001). A vast majority of CRPC patients were castrated until the end of the study, with the cumulative probability of testosterone <50 mg/dL of 91.7% for relugolix and 97% for leuprolide. 

### 3.3. Safety

A summary of adverse events (AEs) for the overall mITT population and the metastatic disease population is shown in Table 3. In the mITT population, the frequencies of AEs overall were similar between relugolix and leuprolide, with no new safety signals observed. Hot flashes (53.8% and 51.0% in the relugolix and leuprolide groups, respectively) and fatigue (22.0% and 19.0%) were the most common AEs in both groups. Patients in the relugolix group reported a higher occurrence of diarrhea (11.4% vs. 6.4%) than in the leuprolide group. There were no patient withdrawals due to diarrhea and all events were mild or moderate (grade 1 or grade 2). 

No new safety signals were observed and the overall frequencies of AEs were similar between relugolix and leuprolide in the metastatic population (Table 3). In the metastatic population, the most common AE was hot flashes in both groups (50.3% and 45.8% in the relugolix and leuprolide groups, respectively). Arthralgia (16.2% vs. 9.0%) and diarrhea (10.0 vs. 4.9%) occurred in a higher proportion of patients in the relugolix group than in the leuprolide group. As in the overall population, all diarrhea events were mild or moderate (grade 1 or grade 2) and no patient was withdrawn due to diarrhea. Back pain was reported in a higher proportion of men in the leuprolide group (15.3%) than in the relugolix group (9.7%); in the overall analysis, back pain was reported in 9.8% of men in the leuprolide group and 8.5% of men in the relugolix group.

The AE profile of men who experienced a CRFS event was similar to the profile for the overall population, with increases in grade ≥3, serious, and AEs in the CRPC population (Table S2).

## 4. Discussion

The oral GnRH receptor antagonist, relugolix and leuprolide demonstrated similar CRFS in men with metastatic disease or in the overall population of the HERO study through 48 weeks. Through 48 weeks of treatment with relugolix, approximately 76.6% of men with metastatic prostate cancer remained castration resistance-free, which was consistent with treatment with leuprolide. These results are better than what has been reported in recent studies of men with untreated mCSPC and suggest that metastatic prostate cancer patients in the HERO study had a more favorable disease burden. More accurate descriptions of tumor burden including number of metastases or cumulative size of measurable metastatic disease should be considered. Nonetheless, many patients with metastatic disease may benefit from combination therapy with an androgen receptor pathway inhibitor as well as GnRH agonist or antagonist therapy. As noted in the primary HERO study publication [13], diarrhea was reported in a higher percentage of patients in the relugolix than the leuprolide group, whereas a lower incidence of MACE was reported in the relugolix group versus the leuprolide group. There were no study withdrawals due to diarrhea and all diarrhea cases were mild or moderate.

We evaluated several baseline characteristics for prognostic significance in this analysis. Baseline testosterone levels (<250 ng/dL, ≥250 ng/dL) were not a risk factor for CRFS progression events and all but 2 patients (both in the leuprolide arm) with a CRFS progression event had testosterone levels below the castration threshold at the time of the event. Baseline PSA ≥ 20 ng/mL (*p* < 0.0001), metastatic disease at baseline (*p* < 0.0001), and former or current smoker (*p* = 0.0284) were independently significant risk factors for CRFS events. The incidence of AEs in the population of men with metastatic disease was generally consistent with that observed in the primary analysis of HERO with no new safety signals observed. As may be expected with a metastatic disease population, the frequency of AEs was higher than the overall population, most likely due to the more advanced stage of their disease. In the primary analysis of the HERO study published in 2020 [13], 96.7% of men receiving relugolix treatment achieved suppression of testosterone to castrate levels, which was superior to leuprolide (88.8%). Relugolix treatment was also associated with a 54% risk reduction in MACE when compared to men receiving leuprolide treatment. Based on the totality of the HERO study data, relugolix was approved by the FDA as the first oral GnRH receptor antagonist for adult men with advanced prostate cancer.

Progression to CRPC is associated with a shortened overall survival and a need for additional therapy [20,21]. In addition, the time to CRPC development has been shown to be shorter in high-risk metastatic prostate cancer patients. Alternative novel treatment modalities are required for these patients [22], with additional hormonal therapies or chemotherapy added to ADT [16,23,24,25]. To date, no single ADT has shown superior CRFS to another ADT regimen. In men in the relugolix group who became castrate resistant, all were under the castration threshold (<50 ng/dL) at the time of their CRFS progression event and >90% were at castrate levels throughout 48 weeks, which shows that men whose prostate cancer became castrate resistant still demonstrate castrate levels of testosterone.

This analysis does have some limitations. The HERO study was conducted over 48 weeks, which may not be a sufficient timeframe to observe CRFS in this patient population. In addition, metastatic patients were not allowed combination therapy at the time of study start (i.e., ADT monotherapy only), which is inconsistent with current guidelines [5,25], although additional treatment was allowed once patients had established CRPC, including enzalutamide or docetaxel. Of note, there may be an issue with medication compliance with oral agents relative to injectables, however, there was a 99% compliance rate for relugolix in the HERO study [13].

## 5. Conclusions

In the HERO study, relugolix demonstrated rapid and sustained suppression of testosterone levels superior to that with leuprolide in men with advanced prostate cancer. The onset of castration-resistance results were similar for relugolix and the previous standard-of-care leuprolide in men with metastatic disease as well as those in the overall HERO mITT population. In this analysis, baseline testosterone levels were not a driver of early castrate resistance. However, PSA > 20, metastatic disease at baseline, and smoking were significant risk factors for a CRFS progression event.

## Figures and Tables

**Table 1:** Baseline Characteristics.

	Overall Population		Metastatic Population	
	Relugolix (N = 717)	Leuprolide (N = 357)	Relugolix (N = 290)	Leuprolide (N = 144)
**Age category**				
≤75 years	509 (71.0%)	254 (71.1%)	205 (70.7%)	101 (70.1%)
>75 years	208 (29.0%)	103 (28.9%)	85 (29.3%)	43 (29.9%)
**Age, years**				
Median	71.0	71.0	71.0	71.0
Min, Max	48, 91	47, 97	48, 91	47, 89
**Geographic region**				
North America	208 (29.0%)	102 (28.6%)	70 (24.1%)	33 (22.9%)
South America	45 (6.3%)	24 (6.7%)	22 (7.6%)	13 (9.0%)
Europe	271 (37.8%)	135 (37.8%)	116 (40.0%)	57 (39.6%)
Asia	155 (21.6%)	86 (24.1%)	62 (21.4%)	36 (25.0%)
Rest of World	38 (5.3%)	10 (2.8%)	20 (6.9%)	5 (3.5%)
**Location of metastasis at study entry**				
Bone only	161 (22.5%)	70 (19.6%)	161 (55.5%)	70 (48.6%)
Lymph node only	40 (5.6%)	24 (6.7%)	40 (13.8%)	24 (16.7%)
Visceral only	8 (1.1%)	3 (0.8%)	8 (2.8%)	3 (2.1%)
Multiple	79 (11.0%)	47 (13.2%)	79 (27.2%)	47 (32.6%)
**PSA**				
PSA ≥ 20	289 (40.3%)	144 (40.3%)	182 (62.8%)	100 (69.4%)
**Laboratory Markers**				
LDH above ULN	43 (6.0%)	32 (9.0%)	25 (8.6%)	22 (15.3%)
ALP above ULN	74 (10.3%)	39 (10.9%)	67 (23.1%)	36 (25.0%)
**Gleason score**				
2–4	0	1 (0.3%)	0	0
5–6	101 (14.1%)	47 (13.2%)	27 (3.8%)	12 (3.4%)
7	255 (35.6%)	137 (38.4%)	77 (10.7%)	37 (10.4%)
8–10	341 (47.6%)	166 (46.5%)	178 (24.8%)	92 (25.8%)
Missing	20 (2.8%)	6 (1.7%)	8 (1.1%)	3 (0.8%)

Abbreviations: ALP = alkaline phosphatase; LDH = lactate dehydrogenase; ULN = upper limit of normal.

**Table 2:** Testosterone Values at the Time of CRFS Progression Events (mITT population).

	Testosterone Values at the Time of CRFS Progression Events	
Testosterone Values (ng/dL)	Relugolix ^a^ (N = 717)	Leuprolide ^a^ (N = 357)
0 to ≤10	33	22 + 4 died
10 to ≤20	43	13 + 1 died
20 to ≤30	7 + 1 died	0
30 to ≤40	1	0
40 to ≤50	3	0
>50	0	2 died ^b^

Abbreviations: CRFS = castration resistance-free survival; mITT = modified intention-to-treat. ^a^ For men who died, the last testosterone values measured before death were used. ^b^ Both patients died with last testosterone >100 ng/dL.

**Table 3:** Adverse Events Summary for the Overall mITT Population and the Metastatic Disease Population.

	Overall Population				Metastatic Population			
	Relugolix (N = 717)		Leuprolide (N = 357)		Relugolix (N = 290)		Leuprolide (N = 144)	
	Any Grade n (%)	Grade ≥ 3 n (%)	Any Grade n (%)	Grade ≥ 3 n (%)	Any Grade n (%)	Grade ≥ 3 n (%)	Any Grade n (%)	Grade ≥ 3 n (%)
Any adverse event	664 (92.6)	136 (19.0)	330 (92.4)	70 (19.6)	268 (92.4)	72 (24.8)	129 (89.6)	35 (24.3)
Serious adverse event	89 (12.4)	—	51 (14.3)	—	49 (16.9)	—	24 (16.7)	—
Fatal adverse event	10 (1.4)	—	11 (3.1)	—	9 (3.1)	—	7 (4.9)	—
Adverse events that occurred in >10% of patients in either group								
Hot flush	386 (53.8)	4 (0.6%)	182 (51.0)	0	146 (50.3)	0	66 (45.8)	0
Fatigue	158 (22.0)	4 (0.6)	68 (19.0)	0	67 (23.1)	2 (0.7)	29 (20.1)	0
Arthralgia	87 (12.1)	2 (0.3)	32 (9.0)	0	47 (16.2)	2 (0.7)	13 (9.0)	0
Constipation	91 (12.7)	0	36 (10.1)	0	45 (15.5)	0	22 (15.3)	0
Hypertension	61 (8.5)	15 (2.1)	37 (10.4)	2 (0.6)	34 (11.7)	10 (3.4)	14 (9.7)	1 (0.7)
Nausea	48 (6.7)	0	16 (4.5)	0	32 (11.0)	0	10 (6.9)	0
Diarrhea	82 (11.4)	0	23 (6.4)	0	29 (10.0)	0	7 (4.9)	0
Back pain	61 (8.5)	3 (0.4)	35 (9.8)	3 (0.8)	28 (9.7)	2 (0.7)	22 (15.3)	3 (2.1)

Abbreviations: MACE, major adverse cardiovascular event; SMQ, standardized MedDRA query. Adverse event grades are evaluated based on the National Cancer Institute Common Terminology Criteria for Adverse Events Version 4.03. MedDRA Version 22.0.

## Data Availability

Data will be made available based on reasonable requests.

## Supplementary Materials

The following supporting information can be downloaded at: https://www.mdpi.com/article/10.3390/cancers15194854/s1, Table S1. Baseline Characteristics of Men with an Event (CRPC population) Compared to the Overall Population; Table S2. Adverse Events Summary for Men with an Event (CRPC population) Compared to the Overall Population.
