# Supplementary material for: Relugolix vs. Leuprolide Effects on Castration Resistance-Free Survival from the Phase 3 HERO Study in Men with Advanced Prostate Cancer
Source: Cancers (Basel). 2023 Oct 5;15(19):4854. doi: 10.3390/cancers15194854 (PMC10571668; doi:10.3390/cancers15194854)
Supplement: Supplementary file 1 [file cancers-15-04854-s001.zip › cancers-2571306-supplementary.pdf]

**Supplemental Tables**

**Table S1.** Baseline Characteristics of Men with an Event (CRPC population) Compared to the Overall Population.

| | Overall Population | | | CRPC Population | | |
|---|---|---|---|---|---|---|
| | Relugolix (N = 717) | Leuprolide (N = 357) | Total (N = 1074) | Relugolix (N = 88) | Leuprolide (N = 42) | Total (N = 1074) |
| **Age category** | | | | | | |
| ≤ 75 years | 509 (71.0%) | 254 (71.1%) | 763 ( 71.0%) | 60 (68.2%) | 26 (61.9%) | 86 ( 66.2%) |
| > 75 years | 208 (29.0%) | 103 (28.9%) | 311 ( 29.0%) | 28 (31.8%) | 16 (38.1%) | 44 ( 33.8%) |
| **Age, years** | | | | | | |
| Median | 71.0 | 71.0 | 71.0 | 72.0 | 71.5 | 72.0 |
| Min, Max | 48, 91 | 47, 97 | 47, 97 | 54, 91 | 57, 87 | 54, 91 |
| **Geographic region** | | | | | | |
| North America | 208 (29.0%) | 102 (28.6%) | 310 ( 28.9%) | 15 (17.0%) | 12 (28.6%) | 27 ( 20.8%) |
| South America | 45 (6.3%) | 24 (6.7%) | 69 ( 6.4%) | 8 (9.1%) | 1 (2.4%) | 9 ( 6.9%) |
| Europe | 271 (37.8%) | 135 (37.8%) | 406 ( 37.8%) | 33 (37.5%) | 20 (47.6%) | 53 ( 40.8%) |
| Asia | 155 (21.6%) | 86 (24.1%) | 241 ( 22.4%) | 26 (29.5%) | 7 (16.7%) | 33 ( 25.4%) |
| Rest of World | 38 (5.3%) | 10 (2.8%) | 48 ( 4.5%) | 6 (6.8%) | 2 (4.8%) | 8 ( 6.2%) |
| **Disease stage at study entry** | | | | | | |
| Metastatic | 290 ( 40.4%) | 144 ( 40.3%) | 434 ( 40.4%) | 68 ( 77.3%) | 32 ( 76.2%) | 100 ( 76.9%) |
| Locally advanced | 192 ( 26.8%) | 96 ( 26.9%) | 288 ( 26.8%) | 12 ( 13.6%) | 7 ( 16.7%) | 19 ( 14.6%) |
| Localized | 178 ( 24.8%) | 83 ( 23.2%) | 261 ( 24.3%) | 8 ( 9.1%) | 1 ( 2.4%) | 9 ( 6.9%) |
| Not classifiable | 57 ( 7.9%) | 34 ( 9.5%) | 91 ( 8.5%) | 0 | 2 ( 4.8%) | 2 ( 1.5%) |
| **Location of metastasis at study entry** | | | | | | |
| Bone only | 161 (22.5%) | 70 (19.6%) | 231 ( 21.5%) | 29 (33.0%) | 14 (33.3%) | 43 ( 33.1%) |
| Lymph node only | 40 (5.6%) | 24 (6.7%) | 64 ( 6.0%) | 7 (8.0%) | 0 | 7 ( 5.4%) |
| Visceral only | 8 (1.1%) | 3 (0.8%) | 11 ( 1.0%) | 0 | 0 | 0 |
| Multiple | 79 (11.0%) | 47 (13.2%) | 126 ( 11.7%) | 31 (35.2%) | 18 (42.9%) | 49 ( 37.7%) |
| **Clinical disease state presentation** | | | | | | |
| Evidence of biochemical (PSA) or clinical relapse following local primary intervention with curative intent | 324 ( 45.2%) | 167 ( 46.8%) | 491 ( 45.7%) | 22 ( 25.0%) | 10 ( 23.8%) | 32 ( 24.6%) |
| Newly diagnosed androgen-sensitive metastatic disease | 219 ( 30.5%) | 109 ( 30.5%) | 328 ( 30.5%) | 56 ( 63.6%) | 27 ( 64.3%) | 83 ( 63.8%) |
| Advanced localized disease not suitable for primary surgical intervention with curative intent | 174 ( 24.3%) | 81 ( 22.7%) | 255 ( 23.7%) | 10 ( 11.4%) | 5 ( 11.9%) | 15 ( 11.5%) |
| **ECOG status** | | | | | | |
| 0 | 619 ( 86.3%) | 307 ( 86.0%) | 926 ( 86.2%) | 69 ( 78.4%) | 28 ( 66.7%) | 97 ( 74.6%) |
| 1 | 98 ( 13.7%) | 49 ( 13.7%) | 147 ( 13.7%) | 19 ( 21.6%) | 13 ( 31.0%) | 32 ( 24.6%) |
| 3 | 0 | 1 ( 0.3%) | 1 ( 0.1%) | 0 | 1 ( 2.4%) | 1 ( 0.8%) |

| | Overall Population | | | CRPC Population | | |
|---|---|---|---|---|---|---|
| | Relugolix (N = 717) | Leuprolide (N = 357) | Total (N = 1074) | Relugolix (N = 88) | Leuprolide (N = 42) | Total (N = 1074) |
| **PSA (ng/mL)** | | | | | | |
| Median | 13.4 | 12.5 | 13.3 | 105.9 | 84.1 | 103.4 |
| Min, Max | 0.2, 10270.0 | 0.2, 17574.0 | 0.2, 17574.0 | 2.8, 7389.0 | 6.7, 17574.0 | 2.8, 17574.0 |
| PSA ≥20 | 289 (40.3%) | 144 (40.3%) | 433 (40.3%) | 71 (80.7%) | 34 (81.0) | 105 (80.5%) |
| **Testosterone (ng/dL)** | | | | | | |
| Mean (SD) | 436.4 (161.5) | 409.0 (152.3) | 427.4 (159.0) | 418.1 (146.7) | 390.7 (139.3) | 409.4 (144.4) |
| **Smoking history** | | | | | | |
| Never smoker | 304 ( 42.4%) | 146 ( 40.9%) | 450 ( 41.9%) | 44 ( 50.0%) | 18 ( 42.9%) | 62 ( 47.7%) |
| Former smoker | 339 ( 47.3%) | 174 ( 48.7%) | 513 ( 47.8%) | 35 ( 39.8%) | 17 ( 40.5%) | 52 ( 40.0%) |
| Current smoker | 74 ( 10.3%) | 37 ( 10.4%) | 111 ( 10.3%) | 9 ( 10.2%) | 7 ( 16.7%) | 16 ( 12.3%) |
| **Laboratory Markers** | | | | | | |
| LDH above ULN | 43 (6.0%) | 32 (9.0%) | 75 (7.0%) | 15 (17.0%) | 14 (33.3%) | 29 (22.3%) |
| ALP above ULN | 74 (10.3%) | 39 (10.9%) | 113 (10.5%) | 30 (34.1%) | 11 (26.2%) | 41 (31.5%) |

Abbreviations: ALP = alkaline phosphatase; CRPC = castrations-resistant prostate cancer; LDH = lactate dehydrogenase; ULN = upper limit of normal.

**Table S2.** Adverse Events Summary for Men with an Event (CRPC population) Compared to the Overall Population.

| | Overall Population | | | | CRPC Population | | | |
|---|---|---|---|---|---|---|---|---|
| | Relugolix (N = 717) | | Leuprolide (N = 357) | | Relugolix (N = 88) | | Leuprolide (N = 42) | |
| | Any Grade n (%) | Grade ≥3 n (%) | Any Grade n (%) | Grade ≥3 n (%) | Any Grade n (%) | Grade ≥3 n (%) | Any Grade n (%) | Grade ≥3 n (%) |
| Any adverse event | 664 (92.6) | 136 (19.0) | 330 (92.4) | 70 (19.6) | 80 (90.9) | 25 (28.4) | 41 (97.6%) | 16 (38.1%) |
| Serious adverse event | 89 (12.4) | — | 51 (14.3) | — | 16 (18.2) | — | 17 (40.5) | — |
| Fatal adverse event | 10 (1.4) | — | 11 (3.1) | — | 4 (4.5) | — | 9 (21.4) | — |
| Adverse events that occurred in >10% of patients in either group | | | | | | | | |
| Hot flush | 386 (53.8) | 4 (0.6%) | 182 (51.0) | 0 | 42 (47.7) | 2 (2.3) | 20 (47.6) | 0 |
| Fatigue | 158 (22.0) | 4 (0.6) | 68 (19.0) | 0 | 24 (27.3) | 1 (1.1) | 12 (28.6) | 0 |
| Arthralgia | 87 (12.1) | 2 (0.3) | 32 (9.0) | 0 | 16 (18.2) | 2 (2.3) | 4 (9.5) | 0 |
| Constipation | 91 (12.7) | 0 | 36 (10.1) | 0 | 18 (20.5) | 0 | 5 (11.9) | 0 |
| Hypertension | 61 (8.5) | 15 (2.1) | 37 (10.4) | 2 (0.6) | 6 (6.8) | 2 (2.3) | 1 (2.4) | 0 |
| Nausea | 48 (6.7) | 0 | 16 (4.5) | 0 | 10 (11.4) | 0 | 5 (11.9) | 0 |
| Diarrhea | 82 (11.4) | 0 | 23 (6.4) | 0 | 4 (4.5) | 0 | 2 (4.8) | 0 |
| Back pain | 61 (8.5) | 3 (0.4) | 35 (9.8) | 3 (0.8) | 8 (9.1) | 1 (1.1) | 4 (9.5) | 1 (2.4) |

Abbreviations: MACE, major adverse cardiovascular event; SMQ, standardised MedDRA query.

[a]Search criteria included Myocardial Infarction SMQ (broad), Central Nervous System Hemorrhages and Cerebrovascular Conditions SMQ (broad), and deaths due to all causes.

[b]Number of patients without a history of MACE was 538 in relugolix group and 263 in leuprolide group.

[c]Number of patients without a history of MACE was 84 in relugolix group and 45 in leuprolide group.

Adverse event grades are evaluated based on National Cancer Institute Common Terminology Criteria for Adverse Events Version 4.03.

MedDRA Version 22.0.
